# Self-Rated Health Status and Subjective Health Complaints Associated with Health-Promoting Lifestyles among Urban Chinese Women: A Cross-Sectional Study

**DOI:** 10.1371/journal.pone.0117940

**Published:** 2015-02-11

**Authors:** Jingru Cheng, Tian Wang, Fei Li, Ya Xiao, Jianlu Bi, Jieyu Chen, Xiaomin Sun, Liuguo Wu, Shengwei Wu, Yanyan Liu, Ren Luo, Xiaoshan Zhao

**Affiliations:** 1 Department of Traditional Chinese Medicine, Nanfang Hospital, Southern Medical University, Guangzhou, Guangdong, 510515, China; 2 School of Traditional Chinese Medicine, Southern Medical University, Guangzhou, Guangdong, 510515, China; 3 Department of Rheumatic Diseases, The First Affiliated Hospital of Guangzhou University of Chinese Medicine, Guangzhou, 510405, China; Weill Cornell Medical College Qatar, QATAR

## Abstract

**Objective:**

This study aimed to investigate whether self-rated health status (SRH) and subjective health complaints (SHC) of urban Chinese women are associated with their health-promoting lifestyles (HPL).

**Methods:**

We conducted a cross-sectional study on 8142 eligible Chinese participants between 2012 and 2013. Demographic and SHC data were collected. Each subject completed the SRH questionnaire and the Chinese version of the Health-Promoting Lifestyle Profile-II (HPLP-II). Correlation and binary regression analyses were performed to examine the associations of SRH and SHC with HPL.

**Results:**

Both SRH and HPL of urban Chinese women were moderate. The most common complaints were fatigue (1972, 24.2%), eye discomfort (1571, 19.3%), and insomnia (1542, 18.9%). Teachers, highly educated subjects and elderly women had lower SRH scores, while college students and married women had better HPL. All items of HPLP-II were positively correlated with SRH (r = 0.127-0.533, *P* = 0.000) and negatively correlated with SHC to a significant extent (odds ratio [OR] = 1.40-11.37).

**Conclusions:**

Aspects of HPL, particularly stress management and spiritual growth, are associated with higher SRH and lower SHC ratings among urban Chinese women. Physical activity and health responsibility are additionally related to reduced fatigue and nervousness. We believe that these findings will be instrumental in encouraging researchers and urban women to adopt better health-promoting lifestyles with different priorities in their daily lives.

## Introduction

China is a nation in transition. In recent years, adverse lifestyle changes that tend to accompany industrialization and urbanization have become increasingly prevalent. Such changes may be associated with adverse health outcomes and poor quality of life [1], and to some extent, contribute to the surprisingly large proportion of sufferers from chronic diseases and associated premature deaths [2, 3]. These problems disproportionately affect women, who account for 48.73% of the total Chinese population [4].

Urban women fulfill multiple social roles and report higher stress and health problems [5, 6], owing to more work, educational and economic pressures than rural women [7] in addition to continued responsibility for their homes and children, resulting in higher total workload and less time to attend to their own needs, compared to men [6]. The female gender is considered a predictor of lower status, lower participation in decision making and lower pay, and women are more likely to be employed in positions that demand a high work pace but allow little opportunity for advancement [8, 9]. Moreover, older aged unmarried girls and divorced women are increasingly common in the urban cities and are subjected to the pressure of overwhelming public opinion on account of, what is considered to be, their embarrassing status in Chinese culture (in which the social role and status of women in society heavily depend on marriage and men) [10]. These physical and psychological strains have a significant influence on women’s health status [11]. Several studies have consistently reported that women are more likely than men to experience health problems, such as insomnia, headache and fatigue [12, 13].

Two important factors associated with 10 major causes of death are unhealthy behavior and lifestyle [14]. Health complaints are critical factors for long-term sick leave [15]. Several studies have been published regarding women’s health status, and the Health-Promoting Lifestyle Profile-II (HPLP-II) is widely used to measure the behavior of women in Western society [14,16]. However, Chinese women have received relatively little research attention to date, and limited studies have explored their self-rated health (SRH) or prevalence of health complaints. Even fewer reports have assessed whether health-promoting lifestyles (HPL) are associated with poor SRH and increased subjective health complaints (SHC). However, women are a keystone for maintaining family health in all cultures, and their health is a fundamental component of development and improvement worldwide [17]. Lack of attention to health in women can adversely affect the health of future generations.

In the present study, the primary objective was to evaluate SRH and explore the prevalence of several SHC among urban Chinese women aged between 20 and 40 years in Guangdong Province. The secondary objective was to ascertain the impact of demographic characteristics and HPL on SRH and the six most common SHC. These studies are necessary for developing health services and interventions for urban women and improving the healthcare system. To our knowledge, no reports to date have focused on the correlations among factors related to SRH and SHC in a cross-section of urban Chinese women.

## Methods

### Participants

Between April 2012 and January 2013, a cross-sectional study was carried out using a self-administered questionnaire while interviewing a population of urban women. The study was conducted in 6 cities (Guangzhou, Huizhou, Shaoguan, Jiangmen, Zhanjiang, and Heyuan) randomly selected from the 21 cities in Guangdong Province. One or two convenient areas in each city were selected for cluster sampling.

Selected participants were women aged 20 to 40 years who met the following inclusion criteria: (i) not pregnant or lactating, (ii) had lived in the urban area for more than one year, and (iii) no critical illness or intake of medication in the previous 2 weeks. To increase participation and completeness/truthfulness of each questionnaire, recruitment was conducted in conference halls of different selected units in cooperation with the administrators. We completed 8279 questionnaires, with a rejection rate of 8.1%. Subjects with missing information on health status, health complaints and HPLP-II (n = 137) were excluded. Overall, this cross-sectional study was performed on 8142 eligible participants (74.0% of the individuals contacted).

### Measurements

The questionnaire included an introduction detailing the objectives of the study and guaranteeing anonymity and confidentiality of data. Four further sections pertained to demographic characteristics, SHC, SRH, and HPLP-II. The section on basic characteristics included information on age, job, marital status, education attainment, body mass index (BMI), and symptoms often experienced by urban women. These symptoms were selected based on related articles in the literature [18,19]. The following questions were answered using a “Yes” or “No” format: (i) are you prone to fatigue and find it difficult to feel energized?, (ii) are you usually subjected to eye discomfort, such as dryness, fatigue, itching, etc., (iii) do you often suffer from insomnia, (iv) do you often have dizziness or headache, (v) are you inclined to experience gastrointestinal upset, and (vi) do you get nervous easily?

Participants were also asked to complete two surveys, including the suboptimal health status questionnaire-39 (SHMS V1.0–39) and Health Promotion Lifestyle Profile-II (HPLP-II).

### Instruments

We previously established the utility of an instrument with Cronbach’s alpha and split-half reliability coefficient of 0.917 and 0.831, respectively (SHMS V1.0) for investigating SRH status [20]. SHMS V1.0 consists of 39 items with 3 dimensions: physical health (14 items), psychological health (12 items), social health (9 items), and 4 other items for subjective health status, whereby participants are asked “what is your general feeling in terms of physical/psychological/ social/general health?” The 35 items of five-point Likert-type (1 = never, 2 = occasionally, 3 = sometimes, 3 = often, 4 = routinely) were used to measure the respondent’s self-reported health problems. Transformed scores were determined to account for reverse questions. The total transformed score of SHMS V1.0 ranged from 0 to 100, with a higher score representing better SRH.

The health-promoting lifestyle profile (HPLP) was initially developed by Walker, Sechrist, and Pender in 1987 [21] and later revised as HPLP-II (1995) [22]. The Chinese version of HPLP-II was developed by Lee and Loke, who established validity and credibility with a Cronbach’s alpha internal consistency coefficient of 0.91 [23]. The scale comprises six subgroups with a total of 52 items, including spiritual growth (9 items), health responsibility (9 items), physical activity (8 items), nutrition (9 items), interpersonal relations (9 items), and stress management (8 items). Each subgroup can be used independently [16]. Higher scores indicate a healthier lifestyle. All items of HPLP-II are affirmative, with no reverse questions. The answers are given within a four-point Likert-type scale, where ratings of “never”, “sometimes”, “frequently”, and “regularly” are scored as 1, 2, 3, and 4 points, respectively. Therefore, the total HPLP-II score ranged between 52 and 208, subscale scores of physical activity and stress management from 8 to 32, and the other four subscales from 9 to 36. For descriptive scores and logistic regression analysis, trichotomies were used to divide HPLP-II ratings into good, moderate and poor groups. For the total HPLP-II scale, we scored 52–104 as “poor”, 105–156 as “moderate”, and 157–208 as “good”. For spiritual growth, health responsibility, interpersonal relations, and nutrition, scores of 9–18 were taken as “poor”, 19–27 as “moderate”, and 28–36 as “good”. With regard to physical activity and stress management, scores of 8–16, 17–24, and 25–32 were “poor”, “moderate”, and “good”, respectively.

### Data analysis

The data were double inputted by trained assistants into Epidata 3.02 (Epidata, Odense, Denmark) and exported to the Statistical Package for Social Sciences 13.0 (SPSS, Inc., Chicago, IL, USA) for analysis. Subject characteristics were summarized as mean ± standard deviation (SD) for continuous variables and count (n) with percentage (%) for categorical variables. SRH and HPLP-II measurements were presented as mean ± SD and range (min-max). Spearman’s, point biserial and point multiserial correlation analyses were performed to determine the coefficients of correlation. Binary logistic regression analysis was additionally utilized to identify SHC while considering subject characteristics and HPL. All statistical data were considered significant at *P*<0.05.

### Ethical Considerations

The study was approved by the Ethics Committee of Nanfang Hospital in Guangzhou, China (2012) LunShenZi (No. 035). Written informed consent was obtained from subjects after the study objectives were explained. The subjects were free to withdraw at any time without providing a reason. Strict confidentiality was maintained throughout the process of data collection and analysis.

## Results

Table 1 shows the demographic characteristics and SHC of the 8142 subjects. Overall, 2770 (34.0%) were college students, while 3282 (40.3%) worked as teachers, 259 (3.2%) as civil servants, and 1831 (22.5%) as factory employees. In terms of SHC, 1972 (24.2%) subjects reported frequent fatigue, 1571 (19.3%) eye discomfort, 1542 (18.9%) insomnia, 1411 (17.3%) gastrointestinal upset, 1014 (12.5%) dizziness or headache, and 993 (12.2%) nervousness/anxiety.

**Table 1:** Sociodemographic characteristics and subjective health complaints of participants.

Variables	Total (n = 8142)
**Age (years)**	
20–29	4794(58.9)
30–40	3348(41.1)
**Education level**	
Compulsory school	553(6.8)
High school graduate	2179(26.8)
University/college degree	5410(66.4)
**Marital status**	
Single/divorced	4032(49.5)
Married	4110(50.5)
**Body mass index**	
BMI<18.5	2056(25.3)
18.5≤BMI<25	5776(70.9)
25≤BMI	310(3.8)
**Job position**	
College student	2770(34.0)
Teacher	3282(40.3)
Civil servant	259(3.2)
Worker	1831(22.5)
**Subjective health complaints**	
Fatigue	1972(24.2)
Eye discomfort^a^	1571(19.3)
Insomnia	1542(18.9)
Gastrointestinal upset	1411(17.3)
Dizziness or headache	1014(12.5)
Nervousness/anxiety	993(12.2)

Data are presented as n (%) for categorical variables.

^a^Including dryness, fatigue, itching.

Measurements obtained from the SRH and HPLP-II questionnaires are summarized in Table 2. The total average score of SRH was 65.74±10.47 (moderate level), with a lowest average score of 62.81±13.02 for the psychological health subscale. SRH is negatively associated with age, body mass index, education level, job position (S1 Table). The outcomes of HPLP measurement showed relatively low scores for both exercise (15.70 ± 4.39) and health responsibility (17.70 ± 4.10). The total mean HPLP-II score was 123.53±20.27, ranging from 52 to 208. Both HPLP-II and total HPLP-II scores in the better SRH groups were significantly higher (t = 21.46–43.02, *P*<0.001). This finding indicates that SRH is positively associated with HPL (S2 Table), as confirmed in S1 Fig. showing that in all job groups, HPL is positively correlated with SRH. Positive Spearman correlation between SRH scales and all six HPLP-II subcategories (r = 0.271–0.533, *P* = 0.000) was observed (Table 3), particularly for stress management and spiritual growth.

**Table 2:** Self-rated health status and health-promoting lifestyle profile-II findings (n = 8142).

Variables	Mean±SD	Range
**Self-rated health status**	65.74±10.47	(0–100)
Physical health	68.64±11.57	(0–100)
Psychological health	62.81±13.02	(0–100)
Social health	65.14±13.11	(0–100)
**Health-promoting lifestyle profiles**		
Spiritual growth	24.59±5.00	(9–36)
Health responsibility	17.70±4.10	(9–36)
Physical activity	15.70±4.39	(8–32)
Interpersonal relations	24.02±4.39	(9–36)
Nutrition	20.96±4.23	(8–32)
Stress management	20.56±3.93	(9–36)
**Total health-promoting lifestyle profile**	123.53±20.27	(52–208)

**Table 3:** Correlation between self-rated health (SRH) status and health-promoting lifestyle profiles (n = 8142).

Variables	Physical health		Psychological health		Social health		SRH	
	r	P-value	r	P-value	R	P-value	r	P-value
**Total health-promoting lifestyle profile**	0.330	0.000***	0.453	0.000***	0.566	0.000***	0.519	0.000***
Spiritual growth	0.319	0.000***	0.504	0.000***	0.559	0.000***	0.533	0.000***
Health responsibility	0.127	0.000***	0.238	0.000***	0.357	0.000***	0.271	0.000***
Physical activity	0.228	0.000***	0.260	0.000***	0.314	0.000***	0.310	0.000***
Interpersonal relations	0.256	0.000***	0.351	0.000***	0.550	0.000***	0.436	0.000***
Nutrition	0.199	0.000***	0.254	0.000***	0.300	0.000***	0.291	0.000***
Stress management	0.378	0.000***	0.439	0.000***	0.481	0.000***	0.506	0.000***

r, coefficient of correlation of self-rated health profiles with health-promoting lifestyle scales using Spearman’s correlation analysis.

***P<0.001, indicates significant correlation between self-rated health profiles and characteristics.

The association between SHC and job position or health-promoting lifestyles was determined using binary logistic regression analysis, as shown in Table 4. Teachers were at a higher risk of fatigue, insomnia, dizziness/headache and nervousness (OR = 2.02, 1.40, 1.16 and 1.68 respectively), while college students complained more frequently of gastrointestinal upset and civil servants of eye discomfort (OR = 1.53, 95% CI: 1.06–2.20). Scores for all items of HPLP-II were negatively correlated with SHC. Participants with a poor stress management lifestyle were 2–11 times more likely to undergo SHC. Moreover, physical activity was important for avoiding fatigue (odds ratio OR = 3.66, 95% confidence interval CI: 2.53–5.30) while spiritual growth was essential to combat nervousness (OR = 4.67, 95% CI: 3.71–5.87) and insomnia (OR = 2.33, 95% CI: 1.92–2.82).

**Table 4:** Binary logistic regression analysis of subject characteristics and health-promoting lifestyle profiles with subjective health complaints (n = 8142).

Variables	Fatigue	Eye discomfort^a^	Insomnia	Gastrointestinal upset	Dizziness or Headache	Nervousness
	OR(95% CI)	OR(95% CI)	OR(95% CI)	OR(95% CI)	OR(95% CI)	OR(95% CI)
**Job position** ^b^						
College student	1	1	1	1	1	1
Teacher	2.02(1.66–2.47)***	0.73(0.59–0.90)**	1.40(1.13–1.72)**	0.91(0.73–1.14)	1.16(0.90–1.51)	1.68(1.30–2.18)***
Civil servant	1.53(1.07–2.20)*	1.53(1.06–2.20)*	1.12(0.77–1.64)	0.63(0.40–0.99)*	0.72(0.44–1.19)	0.95(0.55–1.62)
Worker	1.43(1.15–1.77)**	1.35(1.07–1.69)*	1.35(1.07–1.69)*	0.70(0.55–0.89)**	0.97(0.74–1.27)	1.08(0.80–1.44)
**Health-promoting lifestyle profiles** ^c^						
Spiritual growth						
Good	1	1	1	1	1	1
Moderate	1.87(1.63–2.14)***	1.29(1.13–1.47)***	1.61(1.40–1.86)***	1.37(1.19–1.58)***	1.39(1.18–1.64)***	2.06(1.70–2.50)***
Poor	2.97(2.48–3.55)***	1.78(1.47–2.16)***	2.33(1.92–2.82)***	1.70(1.39–2.09)***	1.83(1.46–2.29)***	4.67(3.71–5.87)***
Health responsibility						
Good	1	1	1	1	1	1
Moderate	2.08(1.30–3.33)**	1.71(1.06–2.75)*	1.48(0.94–2.34)	1.47(0.94–2.31)	1.54(0.88–2.70)	2.34(1.18–4.63)*
Poor	3.35(2.11–5.33)***	2.13(1.33–3.41)**	1.93(1.23–3.03)**	1.64(1.05–2.56)*	1.97(1.13–3.43)*	3.55(1.81–7.00)***
Physical activity						
Good	1	1	1	1	1	1
Moderate	1.74(1.19–2.54)**	1.04(0.75–1.44)	1.16(0.82–1.65)	1.04(0.75–1.46)	1.56(0.70–1.59)	1.90(1.13–3.20)*
Poor	3.66(2.53–5.30)***	1.51(1.10–2.08)*	1.81(1.29–2.54)**	1.53(1.10–2.12)*	1.67(1.12–2.48)*	3.28(1.97–5.47)***
Interpersonal relations						
Good	1	1	1	1	1	1
Moderate	1.53(1.33–1.77)***	1.12(0.97–1.30)	1.47(1.26–1.72)***	1.16(1.00–1.35)*	1.26(1.05–1.51)*	1.67(1.37–2.04)***
Poor	1.74(1.41–2.14)***	1.43(1.15–1.78)**	1.86(1.49–2.32)***	1.18(0.93–1.49)	1.65(1.28–2.14)***	2.65(2.04–3.44)***
Nutrition						
Good	1	1	1	1	1	1
Moderate	1.80(1.39–2.32)***	1.18(0.92–1.51)	1.35(1.03–1.77)*	1.16(0.89–1.50)	1.48(1.06–2.07)*	1.95(1.35–2.82)***
Poor	2.39(1.83–3.12)***	1.40(1.07–1.82)*	1.97(1.50–2.61)***	1.54(1.17–2.02)**	2.15(1.52–3.04)***	3.00(2.05–4.38)***
Stress management						
Good	1	1	1	1	1	1
Moderate	2.44(2.02–2.96)***	1.28(1.08–1.52)**	2.28(1.85–2.82)***	1.57(1.31–1.87)***	1.50(1.20–1.88)***	3.85(2.77–5.36)***
Poor	5.09(4.09–6.33)***	2.09(1.70–2.58)***	4.52(3.57–5.73)***	2.34(1.86–2.92)***	2.89(2.23–3.73)***	11.37(8.02–16.12)***
**Total health-promoting lifestyle profile** ^c^						
Good	1	1	1	1	1	1
Moderate	2.64(1.95–3.58)***	1.25(0.96–1.62)	1.48(0.94–2.34)	1.47(0.94–2.31)	1.47(1.05–2.06)*	3.47(2.12–5.68)***
Poor	5.08(3.69–7.00)***	1.81(1.36–2.40)***	1.93(1.23–3.03)**	1.64(1.05–2.56)*	2.37(1.65–3.39)***	8.38(5.06–13.88)***

OR, odds ratio; CI, confidence interval;

^a^ Including dryness, fatigue, itching.

^b^ Adjusted for subjects’ age, education level, marital status, and body mass index;

^c^ Adjusted for subjects’ age, education level, marital status, body mass index, and job position.

Correlations of subject characteristics with HPLP-II categories are shown in Table 5. The total HPLP-II score correlated positively with educational level and marital status (r = 0.172 and 0.113, both *P* = 0.001) and negatively with age and BMI (r = −0.028 and −0.127, *P* = 0.012 and 0.000, respectively). Job positions ranked in the order of college students, teachers, civil servants and workers were negatively correlated with the total HPLP-II score (r = −0.251, *P* = 0.000). All HPLP-II subcategories were positively correlated with education level. The majority of subcategories (spiritual growth, physical activity, interpersonal relations, stress management) were positively correlated with marital status and negatively correlated with age and BMI.

**Table 5:** Correlation of health-promoting lifestyle profiles with subject characteristics (n = 8142).

	Spiritual growth		Health responsibility		Physical activity		Interpersonal relations		Nutrition		Stress management		Total health-promoting lifestyle profile	
	r	P-value	r	P-value	r	P-value	r	P-value	r	P-value	r	P-value	r	P-value
Age (years)	﹣0.119	0.000***	0.037	0.001**	﹣0.074	0.000***	﹣0.184	0.000***	0.010	0.382	﹣0.189	0.000***	﹣0.127	0.000***
Education level	0.102	0.000***	0.081	0.000***	0.124	0.000***	0.202	0.000***	0.125	0.000***	0.143	0.000***	0.172	0.001***
Marital status	0.094	0.000***	﹣0.026	0.018*	0.092	0.000***	0.228	0.000***	﹣0.016	0.139	0.164	0.000***	0.113	0.001***
Body mass index	﹣0.030	0.006**	0.023	0.039*	﹣0.007	0.539	﹣0.064	0.000***	0.006	0.572	﹣0.060	0.001***	﹣0.028	0.012*
Job position	﹣0.166	0.000***	﹣0.060	0.000***	﹣0.203	0.000***	﹣0.319	0.000***	﹣0.113	0.000***	﹣0.273	0.000***	﹣0.251	0.000***

r, coefficient of correlation of health-promoting lifestyle profiles with marital status derived through point biseral correlation analysis (Single/divorced,1; Married, 2); with job position and education level through point multivariate correlation analysis (college students, 1; teachers, 2; civil servants, 3; workers, 4 and for education level: compulsory school, 1; high school graduate, 2; university/college degree, 3) with age or body mass index through Spearman’s correlation analysis.

*P<0.05

**P<0.01

***P<0.001 indicate significant correlations between health-promoting lifestyle profiles and subject characteristics.

## Discussion

The results of this study showed that urban women in China consider their health status to be moderate and most frequently complain of fatigue, eye discomfort and insomnia. Although earlier studies disclosed no significant relationships between lifestyle choices (e.g., smoking, consuming alcohol, exercising) and health status [24], we found a moderate health-promoting lifestyle level of urban Chinese women that is correlated positively with SRH (Table 3, r = 0.519, *P* = 0.000; S1 Fig.) and negatively with SHC (Table 4) to a significant extent. Notably, women with poor stress management and spiritual growth were at higher risk of the reported symptoms, compared to those with low scores in other subcategories.

Self-rated health (SRH) is a valid, reliable and robust measure of health status, as well as a predictor of mortality and use of health services [25, 26]. This study confirms the poor health status among women [27] and supports the plausibility of harmful effects of role conflict and overload [28]. Many modern women play multiple roles as wives, mothers and employees trying to meet several demands, and struggle to balance their family relationships and career responsibilities. An earlier study reported that both physical and psychosocial work demands are independent predictors of reduced SRH [29]. Consistent with the findings of Li *et al*. [30], civil servants had the highest SRH score in our study, followed by college students and workers, with the lowest scores determined for teachers (S3 Table). This may be due to vocational strain and limited resources for coping [31]. There is widespread belief that teaching-related stress is a serious problem. Our data clearly showed that teachers are indeed at higher risk of subjective symptoms of fatigue, insomnia, and nervousness, compared to college students, workers and civil servants (S4 Table). However, fatigue, which appears to be the most prominent symptom, even in healthy individuals [32], is the most common complaint among urban women in general, and not just teachers. This finding has been reported by several other researchers [13, 19]. Another recent study showed that women are more at risk of work-related fatigue than men [33].

In terms of health-promoting lifestyles, the moderate HPLP-II scores indicate that urban Chinese women have an interest in health-promoting measures but do not always perform them well. Similar results were obtained in another study on Chinese women [34]. Lifestyle has been established as one of the most important factors affecting health [35]. A health-promoting lifestyle, defined as a “multidimensional pattern of self-initiated actions and perceptions that serve to maintain or enhance the level of wellness, self-actualization, and fulfillment of the individual” [21], can decrease the occurrence of disease, lower the death rate and contribute to improved health status [36–37]. Here, we observed a significant positive relationship between health-promoting lifestyles and SRH among women in all job groups. Furthermore, all items in HPLP-II correlated negatively with health complaints, indicating that high-quality health-promoting lifestyle behaviors lead to fewer health issues. These findings suggest that women need better health-promoting lifestyles to protect against poor health status and complaint symptoms. Lifestyle intervention has been shown to improve fatigue, pain and mental symptoms [32, 38]. Further analyses disclosed that teachers with relatively better consciousness of carrying out health-promoting behaviors, except spiritual growth and stress management, had poorest SRH and were at highest risk of complaint symptoms, while the opposite was observed for civil servants with relatively poor health-promoting behaviors, except stress management and spiritual growth (S3 Table). Based on these results, we propose that spiritual growth and stress management are critical to maintain good health status.

There is clear evidence that chronic stress is associated with elevated blood pressure [39], cardiovascular disease [40], insulin resistance [41] and immunological impairment [42]. However, it is suggested that it is not the presence of stressors but the inability to cope effectively in stressful situations that has unfavorable consequences for health status. Consistently, our results indicate that women performing poorly in stress management are at higher risk of symptom reporting, compared with those performing poorly in other categories of HPLP. Participants in stress management programs have been reported to display significantly fewer symptoms of depression, anxiety, stress, and improvement in sleep quality [43]. Thus, the importance of stress management programs, such as employing the services of stress consultants, should be highlighted among women in urban China.

Spiritual growth reflects the potential of individuals to fulfill themselves in order “to be the best that they are capable of doing”. Expression of one’s creativity, quest for spiritual enlightenment, pursuit of knowledge, and the desire to give to society are examples of self-actualization. An earlier study investigating the relationship between spiritual growth and response to stress among 144 nursing students disclosed that low spiritual growth is a weak predictor of trait anxiety [44]. Additionally, spiritual growth was positively and significantly related to the degree of professionalism. Spiritual growth and stress management are psychological protective factors. In the present study, the risk of poor spiritual growth for each symptom was presented in terms of odds ratios. We found that second only to poor stress management, women with poor spiritual growth had a higher odds ratio of reporting symptoms, with the exception of fatigue and eye discomfort.

Our findings suggest that urban Chinese women are physical inactivity and health irresponsibility (Table 2). However, health responsibility and physical activity were additionally identified as important factors for avoiding fatigue, similar to a recent study [45]. Wessely *et al*. [46] suggested that persistent inactivity as a result of symptoms like pain and fatigue can lead to reduced physiological exercise capacity and the subsequent appearance of more symptoms. Therefore, correct training (working out) and fitness regimes are obviously important factors affecting health in urban women. Moreover, it would be useful for women to take responsibility for their own health, such as booking appointments to receive regular physical examinations and consultations with their doctor.

Lower educational levels are associated with the incidence of coronary heart disease and stroke among women in Europe and the United States [47,48]. Previous studies have shown a higher prevalence of low self-rated health in women with low educational levels [49, 50]. However, results from the current study suggested that high education level is correlated with significantly poor SRH and health complaints (S1 Table). The same conclusion was reached by Sun *et al*. [51] from a study on health status in Guangdong. This discrepancy may be explained by the different study populations. Most of the related literature has focused on the general population or a particular patient subset [49–50]. Our study participants were urban youngadult women (aged 20–40) in China, who contribute significantly to the labor force that has led to a dramatic transformation in social and economic conditions over recent years. In China, educated women are likely to work more often under high time pressure constraints with considerable emotional demands [45]. Higher educated people living in Chinese cities have been reported to experience more stress [2]. In addition to their caregiver roles in the family, their total workloads appear somewhat greater and more diffusely distributed. Moreover, higher education has been associated with health risks associated with work and school, as well as exposure to electromagnetic radiation at work. This is especially prevalent for individuals working with outdated computers and video monitors [52].

### Limitations

Our study has a number of limitations. Firstly, this was a cross-sectional design, which did not allow assessment for the causality or directionality of relationships. Secondly, all information was obtained from self-reported questionnaires, which could result in potential information bias. Consequently, the findings should be cautiously generalized to other populations of women. Further studies on multiple assessments and informants are warranted to provide a more thorough understanding of the relationship between HPLP and SRH and subjective health complaints.

## Conclusions

SRH and HPL status were moderate among urban Chinese women. All items (particularly stress management and spiritual growth) of HPLP-II were positively associated with SRH and negatively correlated with development of SHC. There is an urgent need for improved access to more health-promoting services for urban women, including better stress management, spiritual growth skills and daily physical activity, which are important factors in avoiding fatigue and reducing nervousness and insomnia.

## Supporting Information

S1 Fig Relationship between self-rated health status and HPLP-II score among the four job categories. For all job positions, HPL was positively correlated with SRH.
(TIF)

S1 Table Correlation of self-rated health status with subject characteristics (N = 8142). (DOCX)

S2 Table Measurement of health-promoting lifestyle profiles (HPLP) for (very) good and less good SRH status for all 8142 subjects. (DOCX)

S3 Table Measurement of self-rated health status (SRH) and health-promoting lifestyle profiles (HPLP) in relation to job positions. (DOCX)

S4 Table Subjective health complaints (SHC) among different job positions (N = 8142). (DOCX)
