# Supplementary figures and images for: Self-Rated Health Status and Subjective Health Complaints Associated with Health-Promoting Lifestyles among Urban Chinese Women: A Cross-Sectional Study
Source: PLoS One. 2015 Feb 11;10(2):e0117940. doi: 10.1371/journal.pone.0117940 (PMC4324778; doi:10.1371/journal.pone.0117940)

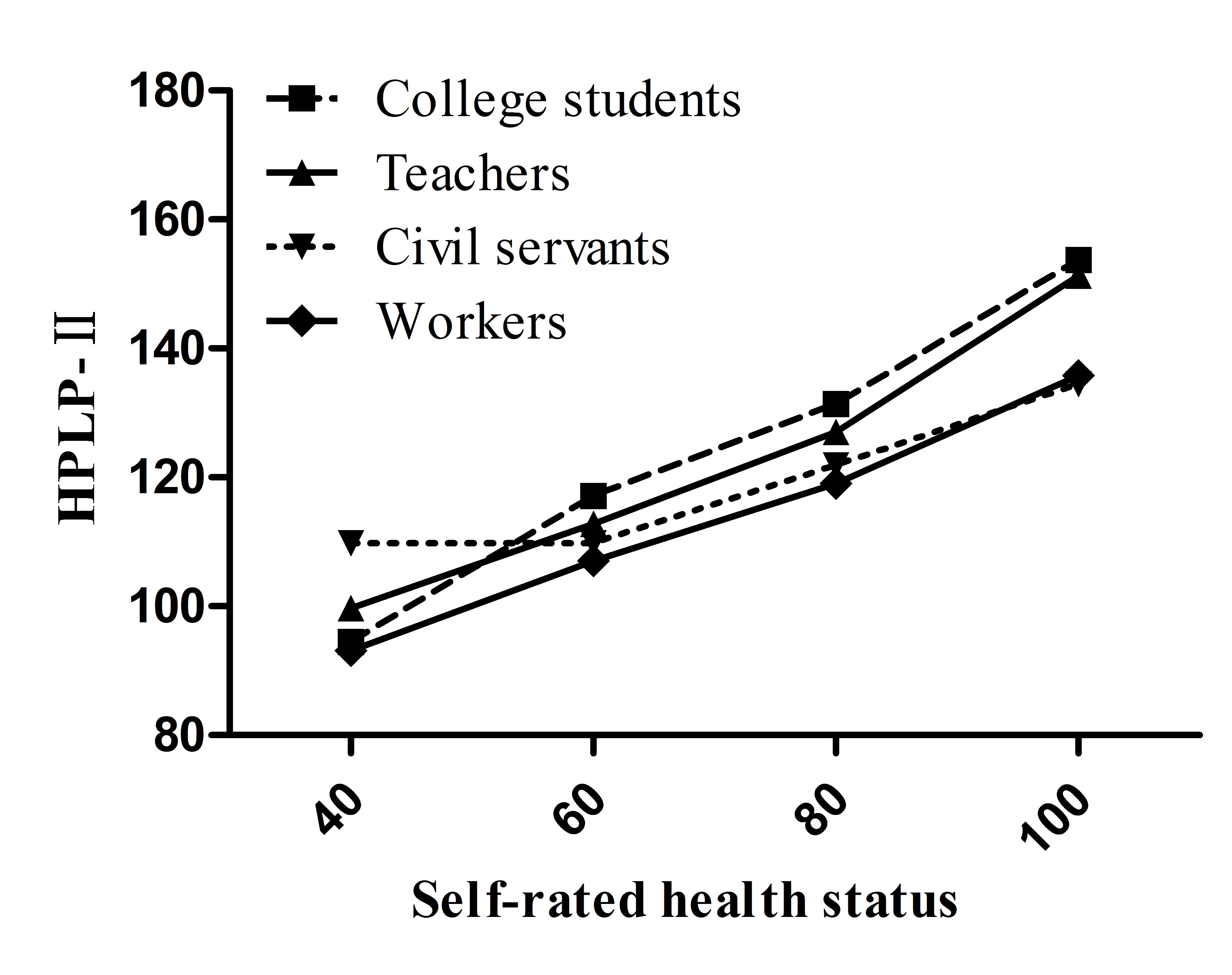

Supplement: S1 Fig — For all job positions, HPL was positively correlated with SRH. (TIF) [file pone.0117940.s001.tif]
