# Supplementary material for: Self-Rated Health Status and Subjective Health Complaints Associated with Health-Promoting Lifestyles among Urban Chinese Women: A Cross-Sectional Study
Source: PLoS One. 2015 Feb 11;10(2):e0117940. doi: 10.1371/journal.pone.0117940 (PMC4324778; doi:10.1371/journal.pone.0117940)
Supplement: S1 Table — (DOCX) [file pone.0117940.s002.docx]

| **Table S1 Correlation of self-rated health status with subject characteristics (N = 8142)** | | | | | | | | |
| --- | --- | --- | --- | --- | --- | --- | --- | --- |
| **Variables** | **Physical health** | | **Psychological  health** | | **Social  communication** | | **SRH** | |
|  | r | P-value | r | P-value | r | P-value | r | P-value |
| Job position | ﹣0.072 | 0.000*** | 0.031 | 0.006** | ﹣0.041 | 0.000*** | ﹣0.057 | 0.000*** |
| Age(years) | ﹣0.174 | 0.000*** | ﹣0.003 | 0.805 | ﹣0.024 | 0.033* | ﹣0.093 | 0.000*** |
| Education level | ﹣0.047 | 0.000*** | ﹣0.085 | 0.000*** | 0.048 | 0.000*** | ﹣0.045 | 0.000*** |
| Marital status | 0.134 | 0.000*** | ﹣0.057 | 0.000*** | 0.038 | 0.001** | 0.047 | 0.000*** |
| Body mass index | ﹣0.055 | 0.000*** | 0.007 | 0.556 | ﹣0.014 | 0.206 | ﹣0.028 | 0.013* |
| r, coefficient of correlation of Self-rated health profiles with Marital and Chronic diseases was derived through the point biserial correlation analysis (Single/divorced,1;Married,2); with job position and education level through the point multiserial correlation analysis (college students, 1; teachers, 2; civil servants, 3;workers, 4.and for education level: Compulsory school,1;High school graduate,2; University/ college degree,3); with age or with body mass index through Spearman’s correlation analysis. *P < 0.05,**P < 0.01, ***P < 0.001, indicate significant correlation between Self-rated health profiles and characteristics. | | | | | | | | |
