# Supplementary material for: Self-Rated Health Status and Subjective Health Complaints Associated with Health-Promoting Lifestyles among Urban Chinese Women: A Cross-Sectional Study
Source: PLoS One. 2015 Feb 11;10(2):e0117940. doi: 10.1371/journal.pone.0117940 (PMC4324778; doi:10.1371/journal.pone.0117940)
Supplement: S2 Table — (DOCX) [file pone.0117940.s003.docx]

| **Table S2 Measurement of health-promoting lifestyle profiles (HPLP) for (very) good and less good SRH status for all 8142 subjects^a^** | | | | | | |
| --- | --- | --- | --- | --- | --- | --- |
| **Variables** | **Total** | **SRH^b^** | | **Range** | t | P |
|  |  | (very) good | less than good |  |  |  |
| **Health-promoting lifestyle profiles** |  |  |  |  |  |  |
| Spiritual growth | 24.59±5.00 | 26.87±4.61 | 22.56±4.43 | 9-36 | 43.02 | 0.000*** |
| Health responsibility | 17.70±4.10 | 18.72±4.32 | 16.80±3.68 | 9-36 | 21.46 | 0.000*** |
| Physical activity | 15.70±4.39 | 16.92±4.50 | 14.60±3.89 | 8-32 | 24.45 | 0.000*** |
| Interpersonal relations | 24.02±4.39 | 25.66±4.35 | 22.56±3.87 | 9-36 | 33.78 | 0.000*** |
| Nutrition | 20.96±4.23 | 22.04±4.40 | 19.99±3.83 | 8-32 | 22.33 | 0.000*** |
| Stress management | 20.56±3.93 | 22.23±3.83 | 19.06±3.37 | 9-36 | 39.56 | 0.000*** |
| **Total health-promoting lifestyle profile** | 123.53±20.27 | 132.44±20.02 | 115.56±16.89 | 52-208 | 40.85 | 0.000*** |
| ^a^Data are represented as mean±SD and range(min.- max.)  ^b^ SRH were divided into two groups by the median SRH score of 66.43: (very)good (SRH score ≥66.43) and less than good(SRH score ＜66.43). ***P＜0.001, indicate significant difference of health-promoting life-style profiles scores among (very) good and less than good SRH | | | | | | |
