# Supplementary material for: Self-Rated Health Status and Subjective Health Complaints Associated with Health-Promoting Lifestyles among Urban Chinese Women: A Cross-Sectional Study
Source: PLoS One. 2015 Feb 11;10(2):e0117940. doi: 10.1371/journal.pone.0117940 (PMC4324778; doi:10.1371/journal.pone.0117940)
Supplement: S3 Table — (DOCX) [file pone.0117940.s004.docx]

| **Table S3 Measurement of self-rated health status (SRH) and health-promoting lifestyle profiles (HPLP) in relation to job positions^a^** | | | | | | | | | | | | |
| --- | --- | --- | --- | --- | --- | --- | --- | --- | --- | --- | --- | --- |
| **Job position** | **SRH** | | | |  | **HPLP** | | | | | | |
|  | **Total SRH** | **Physical  health** | **Psychological  health** | **Social  communication** |  | **Total HPLP** | **Spiritual  growth** | **Health responsibility** | **Physical  activity** | **Interpersonal relations** | **Nutrition** | **Stress  management** |
| College student | 67.05±9.48 | **70.94±10.17** | 62.81±11.74 | **66.65±12.79** |  | **129.11±18.84** | **25.70±4.62** | 17.80±4.20 | **16.67±4.37** | **25.73±4.06** | 21.26±4.15 | **21.96±3.57** |
| Teacher | 64.45±11.36 | 66.17±12.67 | 62.13±13.68 | 64.87±13.81 |  | 123.19±20.84 | 24.26±5.25 | **18.08±4.18** | 15.71±4.44 | 23.69±4.41 | **21.34±4.25** | 20.10±4.05 |
| Civil servant | **67.81±9.26** | 70.84±10.81 | **65.86±12.59** | 65.71±11.46 |  | 119.96±18.41 | 24.72±4.46 | 17.01±3.52 | 14.73±4.13 | 22.98±3.96 | 20.41±4.06 | 20.13±3.78 |
| Worker | 65.78±10.12 | 69.26±10.71 | 63.59±13.58 | 63.30±12.27 |  | 116.19±18.99 | 23.49±4.84 | 16.99±3.78 | 14.34±3.97 | 22.19±3.93 | 19.88±4.17 | 19.31±3.61 |
| ^a^Data are represented as mean±SD. Bold figures means the highest scores compared to other job positions. | | | | | | | | | | | | |
