# Supplementary material for: Self-Rated Health Status and Subjective Health Complaints Associated with Health-Promoting Lifestyles among Urban Chinese Women: A Cross-Sectional Study
Source: PLoS One. 2015 Feb 11;10(2):e0117940. doi: 10.1371/journal.pone.0117940 (PMC4324778; doi:10.1371/journal.pone.0117940)
Supplement: S4 Table — (DOCX) [file pone.0117940.s005.docx]

| **Table S4 Subjective health complaints (SHC) among different job positions (N=8142)^a^** | | | | | | | |
| --- | --- | --- | --- | --- | --- | --- | --- |
| **Self-reported health complaints** | **Total** | **College   student** | **Teacher** | **Civil servant** | **Worker** | **Χ^2^** | **P** |
| Fatigue | **1972(24.2)** | 497(17.9) | **1082(33.0)** | 49(18.9) | 344(18.8) | 229.72 | 0.000*** |
| Eyes-indisposed | 1571(19.3) | **600(21.7)** | 584(17.8) | 50(19.3) | 337(18.4) | 15.634 | 0.001** |
| Insomnia | 1542(18.9) | 457(16.5) | **696(21.2)** | 42(16.2) | 347(19.0) | 22.993 | 0.000*** |
| Gastrointestinal upset | 1411(17.3) | **590(21.3)** | 588(17.9) | 26(10.0) | 207(11.3) | 87.254 | 0.000*** |
| Dizziness or Headache | 1014(12.5) | 274(9.9) | **508(15.5)** | 21(8.1) | 211(11.5) | 50.154 | 0.000*** |
| Nervousness/Anxiety | 993(12.2) | 272(9.8) | **555(16.9)** | 18(6.9) | 148(8.1) | 118.309 | 0.000*** |
| ^a^Data are represented as n(%). Bold figures means the highest SHC rates compared to other job positions. **P＜0.01,***P＜0.001, indicate significant difference of self-reported health complaints rates among job positions. | | | | | | | |
